# Prognostic Impact of del(17p) and del(22q) as Assessed by Interphase FISH in Sporadic Colorectal Carcinomas

**DOI:** 10.1371/journal.pone.0042683

**Published:** 2012-08-17

**Authors:** María González-González, Luís Muñoz-Bellvis, Carlos Mackintosh, Celia Fontanillo, M. Laura Gutiérrez, M. Mar Abad, Oscar Bengoechea, Cristina Teodosio, Emilio Fonseca, Manuel Fuentes, Javier De Las Rivas, Alberto Orfao, José María Sayagués

**Affiliations:** 1 Servicio General de Citometría, Departamento de Medicina and Centro de Investigación del Cáncer (IBMCC-CSIC/USAL), Hospital Universitario de Salamanca-IBSAL, Universidad de Salamanca, Salamanca, Spain; 2 Unidad de Cirugía Hepatobiliopancreática, Departamento de Cirugía, Hospital Universitario de Salamanca-IBSAL, Salamanca, Spain; 3 Centro de Investigación del Cáncer (IBMCC-CSIC/USAL), Universidad de Salamanca, Salamanca, Spain; 4 Grupo de Investigación en Bioinformática y Genómica Funcional, Centro de Investigación del Cáncer (IBMCC-CSIC/USAL), Universidad de Salamanca, Salamanca, Spain; 5 Departamento de Patología, Hospital Universitario de Salamanca-IIBSAL, Salamanca, Spain; 6 Departamento de Oncología Médica, Hospital Universitario de Salamanca-IBSAL, Salamanca, Spain; Howard University, United States of America

## Abstract

**Background:**

Most sporadic colorectal cancer (sCRC) deaths are caused by metastatic dissemination of the primary tumor. New advances in genetic profiling of sCRC suggest that the primary tumor may contain a cell population with metastatic potential. Here we compare the cytogenetic profile of primary tumors from liver metastatic versus non-metastatic sCRC.

**Methodology/Principal Findings:**

We prospectively analyzed the frequency of numerical/structural abnormalities of chromosomes 1, 7, 8, 13, 14, 17, 18, 20, and 22 by iFISH in 58 sCRC patients: thirty-one non-metastatic (54%) *vs.* 27 metastatic (46%) disease. From a total of 18 probes, significant differences emerged only for the 17p11.2 and 22q11.2 chromosomal regions. Patients with liver metastatic sCRC showed an increased frequency of del(17p11.2) (10% *vs.* 67%;p<.001) and del(22q11.2) (0% *vs.* 22%;p = .02) versusnon-metastatic cases. Multivariate analysis of prognostic factors for overall survival (OS) showed that the only clinical and cytogenetic parameters that had an independent adverse impact on patient outcome were the presence of del(17p) with a 17p11.2 breakpoint and del(22q11.2). Based on these two cytogenetic variables, patients were classified into three groups: low- (no adverse features), intermediate- (one adverse feature) and high-risk (two adverse features)- with significantly different OS rates at 5-years (p<.001): 92%, 53% and 0%, respectively.

**Conclusions/Significance:**

Our results unravel the potential implication of del(17p11.2) in sCRC patients with liver metastasis as this cytogenetic alteration appears to be intrinsically related to an increased metastatic potential and a poor outcome, providing additional prognostic information to that associated with other cytogenetic alterations such as del(22q11.2). Additional prospective studies in larger series of patients would be required to confirm the clinical utility of the new prognostic markers identified.

## Introduction

Metastatic dissemination of the primary tumor is the major cause of death of sporadic colorectal cancer (sCRC) patients [1]. Metastasis is a complex multi-step process which is driven by sequential accumulation of multiple genetic and molecular alterations and/or epigenetic changes involving one or multiple tumor cell clones. In recent years, data accumulated about the intratumoral pathways of clonal evolution of sCRC associated with chromosomal alterations/instability, indicates that liver metastatic lesions may derive from descendants of a tumor cell clone which is already present in the primary tumor [2]. Advances in genetic profiling of cancer also suggest that the metastatic potential of human tumors is encoded in the bulk of a primary tumor, as metastatic tumors systematically contain those genetic abnormalities observed in the primary tumor sample from the same subject. However, the precise molecular changes associated with the development of sCRC with liver metastasis still remain to be identified [2]. Multiple recurrent chromosomal abnormalities that are found in primary tumours have been associated with metastatic CRC, including gains of chromosomes 8q, 13q and 20q and losses of the 1p, 8p, 17p, 18q and 22q chromosomal regions [3]–[5].

In a recent study, we described a detailed map of the genetic abnormalities of primary tumors from sCRC patients with liver metastasis by high-resolution SNP arrays. In this study, we reported the existence of a highly prevalent breakpoint region in the great majority of primary sCRC patients who had synchronous liver metastasis. Such breakpoint region is located in the centromeric region of chromosome 17p, between the genome coordinates 20,156,497 bp and 22,975,771 bp [6]. This breakpoint region has been previously associated with i) a homogeneous genetic profile consisting of a higher frequency of abnormalities of chromosomes 1p, 7, 8, 13q, 17p, 18q, 20q and 22q and ii) an adverse clinical outcome [7]. However, delineation of the minimal common breakpoint region at chromosome 17p11.2 and its potential prognostic value in sCRC tumors, remain to be fully defined.

In the present study we investigated the prognostic value of structural/numerical abnormalities of the most frequently altered chromosomes in liver metastatic colorectal carcinomas from 58 sCRC patients (27 liver metastatic *vs.* 31 non-metastatic tumors) with a long median follow-up, as detected by interphase fluorescence *in situ* hybridization (iFISH). Overall, our results show that the occurrence of del(17p) involving the 17p11.2 breakpoint region is an independent prognostic factor for overall survival, as confirmed in a larger series of 119 patients from the GEO public database. However, we have demonstrated that the combined assessment of del(22q11) and del(17p11.2) increased the predictive value for a liver metastatic tumor.

## Materials and Methods

### Patients and samples

In the present study, we prospectively analyzed surgical specimens from 58 patients diagnosed with a sCRC between 1999 and 2010 (38 males and 20 females; median age of 69 years, ranging from 38 to 83 years) after informed consent was given by each subject. All patients underwent surgical resection of primary tumor tissues at the Department of Surgery of the University Hospital of Salamanca (Salamanca, Spain) and they were diagnosed and classified according to the WHO criteria [8] prior to any treatment was given. Fourteen primary tumors were localized in the rectum and the other 44 were localized either in the right (caecum, ascending or trasverse) or the left (descending and sigmoid) colon, with an overall mean size of 5.3±2 cm. According to tumor grade, 39 cases were classified as well-differentiated tumors, 15 as moderately- and four as poorly-differentiated carcinomas. In all cases, histopathological grade was confirmed in a second independent evaluation by an experienced pathologist. Median follow-up at the moment of closing this study was of 96 months (range: 12–124 months). The study was approved by the local ethics committee of the University Hospital of Salamanca (Salamanca, Spain) and informed consent was given by each individual, prior to entering the study.

From the 58 cases analyzed, 27 (47%) tumors had liver metastases (group 1; median follow-up of 37 months; pT3–4 pN1–2 M1) identified either at time of colorectal surgery (n = 16) or during the first year after initial diagnosis (n = 11); they all underwent complete surgical resection of both their primary and metastatic CRC. The other 31 (53%) patients corresponded to non-metastatic sCRC selected on the basis of a long follow-up in the absence of liver metastasis (median follow-up of 99 months; pT2–4 pN0 M0) to ensure their non-metastatic nature (group 2).

After histopathological diagnosis was established, part of the primary tumor was used to prepare single-cell suspensions. Once prepared, single cell suspensions were resuspended in methanol/acetic (3/1; vol/vol) and stored at −20°C for further iFISH analyses, as described elsewhere [2]. The remaining tissue was either fixed in formalin and embedded in paraffin, or frozen in liquid nitrogen and stored at room temperature (RT) and at −80°C, respectively. Each individual tissue sample was also evaluated after haematoxylin-eosin staining, to confirm the presence of tumor cells and to evaluate their quantity.

### Interphase fluorescence *in situ* hybridization (iFISH) studies

**Figure 1:**
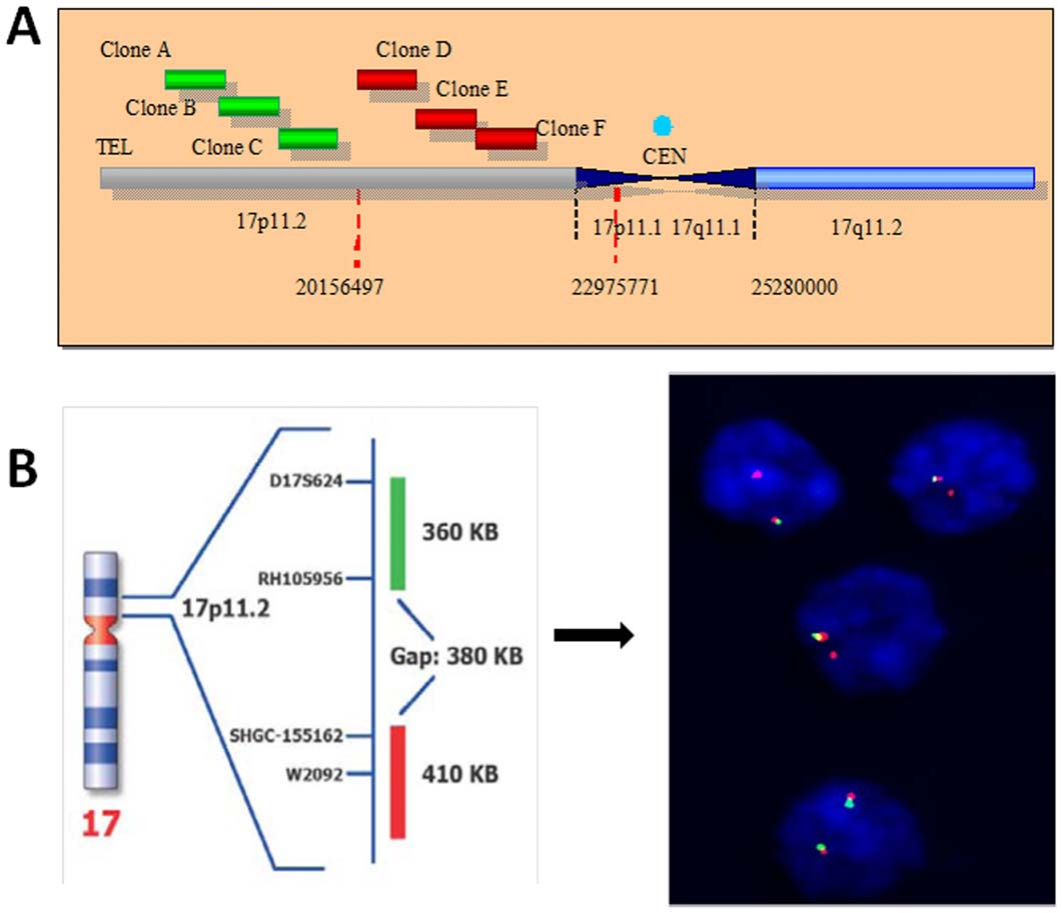
Schematic representation of the chromosome 17p11.2 dual color Break Apart probe combination designed and used for iFISH analysis of this chromosomal region in sCRC. Panel A describes the probe design for which three different clones (A, B and C) directly-labelled with PlatinumBright495 (green signal) and that hybridize to the telomeric part of the 20,156,497 bp region were combined with another three clones (clones D, E and F) directly labelled with PlatinumBright550 and that correspond to sequences harboured centromerically to 20,156,497 bp (red signal), and were produced. The 17p11.2 Break Apart DNA Probe finally consisted of a dual-color assay to detect breakpoints at 17p11.2 using the combination of these 6 fluorescently labelled clones. A positive breakpoint at chromosome 17p11.2 was defined when one or two red/green or yellow fusion signals split into two separate red and green signals. Only red and green signals which were more than one signal diameter apart from each other were counted as reflecting a chromosome break, since based on the probe design a gap of 380 KB exists between the two sets of probes corresponding to the green and the red signals, respectively; two fusion signals identify the two normal chromosomes 17 as illustrated for the lower nuclei shown in panel B. Loss of a green signal in the presence of a single red signal and a fusion signal was interpreted as associated with del(17p) with a 17p11.2 breakpoint (e.g; three upper nuclei in panel B).

Mixed single-cell suspensions from different samples obtained from each tumor were used for iFISH studies, after fixation in 3/1 methanol/acetic (vol/vol). A set of 18 different probes (Vysis Inc, Downers Grove, IL) specific for those chromosomes and chromosomal regions most frequently gained/amplified and deleted colorectal carcinomas with liver metastases [6], were systematically used in double and triple staining with the Spectrum Orange (SO), Spectrum Green (SG) and Spectrum Aqua (SA) fluorochromes: for chromosome 1, the LSI p58 (1p36) (SO)/TelVysion 1p (SG)/LSI 1q25 (SA) Multi-color probe was employed; for chromosome 7, the LSI D7S486 (7q31) (SO)/CEP 7 (SG) Dual Color probe was used; for chromosome 8, the LSI LPL (8p22) (SO)/CEP 8 (SA)/MYC (8q24) (SG) Multi-color probe was employed; for chromosome 13, the LSI RB1 13q14 (SO)/LSI 13q34 LAMP1 (SG) was used; for chromosome 14, the LSI IGH (14q32.33) Dual Color, Break Apart probe was selected; for chromosome 17, the LSI TP53 (17p13) (SO)/CEP 17 (SA) probe combination was employed; for chromosome 18, the LSI BCL2 (18q21) (SO)/CEP 18 (SA) probe combination was used; for chromosome 20, the LSI ZNF217 (20q13.2) (SO)/CEP 20 (SG) probes were employed, and; for chromosome 22, the LSI BCR (22q11.2) probe was used. We have previously found in primary tumors [6] and their paired liver metastases [9] a high prevalence of gains of chromosomes 7, 8q, 11q, 13q, 20q and X together with losses of the 1p, 8p, 17p and 18q chromosomal regions; in this series of cases, the breakpoints found at the centromeric region of chromosome 17p were variable and were mapped between the genomic coordinates 20,156,497 bp and 22,975,771 bp by SNP's arrays. Herein, we investigated the presence of breakpoints at chromosome 17p11.2 using iFISH probes specifically designed and manufactured for this purpose (Kreatech Diagnostics, Amsterdam, The Netherlands), as schematically described in Figure 1.

The specific methods and procedures used for the iFISH studies have been previously described in detail [2] and for the investigation of the relationships existing between those genes coded at the 17p11.2, 17p13.1 and 22q11.2 chromosomal regions and other genes, the Ingenuity Pathway Analysis software (Ingenuit System®,www.ingenuity.com) was used.

### External validation of the prognostic impact of del(17p) and del(22q)

External validation of the prognostic impact of del(17p) and del(22q) was performed in a previously reported group of sCRC patients from which aCGH files (MHP Human 1 Mb) and clinical data were publicly available at the GEO database (accession number GSE12520; genomic markers that predict survivorship in colorectal cancer) [10]. From all cases available in the dataset, we selected those studied with the MHP Human 1 Mb CGH array platform for a total of 109 cases: 81 sCRC from Edinburgh (Scotland, UK) and 38 from Hong Kong. Gpr files were pre-processed and normalized as described elsewhere [11]. Patients included in this external validation group were classified according to the Duke's staging system as follows: stage A, 7.5% (n = 8), B, 44.9% (n = 48), C, 39.2% (n = 42) and stage D (metastatic), 8.5% (n = 9). Median of follow up of these patients was 67 months, with a median overall survival of 28.7months (range: 0.3–147.2 months).

### Statistical methods

For all continuous variables, mean values and their standard deviation (SD) and range were calculated using the SPSS software package (SPSS 15.0 Inc, Chicago, IL USA); for dichotomic variables, frequencies were reported. In order to evaluate the statistical significance of differences observed between groups, the Student's T and the Mann-Whitney U tests were used for continuous variables, depending on whether they displayed or not a normal distribution, respectively. For qualitative variables, the X^2^ test was applied (cross-tab; SPSS). Overall survival (OS) curves were plotted according to the method of Kaplan and Meier, and the log-rank test (one-sided) was used to establish the statistical significance of the differences observed between survival curves (survival; SPSS). Multivariate analysis of prognostic factors for OS was performed using the Cox stepwise regression (forward selection) model (regression, SPSS). For multivariate analysis only those variables showing a significant association with OS in the univariate analysis were included. Statistical significance was considered to be present once *P* values (or, where appropriate, Pearson-corrected *P* values) were <.05.

## Results

### Clinical and biological characteristics of liver metastatic versus non-metastatic sporadic colorectal carcinoma (sCRC)

Overall, sCRC cases with liver metastases showed a higher frequency of lymph node metastases (p≤.001) and abnormally increased CEA serum levels (p≤.001) than non-metastatic patients (Table 1). From the prognostic point of view, sCRC with liver metastases also showed a higher frequency of deaths in association with a significantly shortened patient overall survival (median of 25 months *vs.* not reached, respectively; p≤.001). By contrast, no significant differences were found between liver metastatic *vs.* non-metastatic CRC cases, regarding patient age, gender, tumor localization, histological grade and size, and alkaline phosphatase serum levels (Table 1).

**Table 1:** Clinical and biological characteristics of liver metastatic (n = 27) versus non-metastatic (n = 31) sporadic colorectal carcinoma (sCRC) patients.

	Liver metastatic sCRC (n = 27)	Non-metastatic sCRC (n = 31)	*p*-value	Total cases (n = 58)
**Age** (years)*	73 (48–80)	72 (38–83)	NS	72 (38–83)
**Gender**				
F	11 (41%)	9 (29%)	NS	20 (34%)
M	16 (59%)	22 (71%)		38 (66%)
**Tumor Localization**				
Rectum	5 (19%)	11 (36%)		16 (28%)
Left colon	13 (48%)	15 (48%)	*NS*	28 (52%)
Right colon	9 (33%)	5 (16%)		14 (20%)
**Histological grade**				
Well-differentiated	16 (59%)	23 (74%)		39 (67%)
Moderate-differentiated	8 (30%)	7 (22%)	NS	15 (26%)
Poorly-differentiated	3 (11%)	1(4%)		4 (7%)
**Histopathology**				
pN0	7 (26%)	31 (100%)		38 (66%)
pN1	12 (44%)	0 (0%)	*p≤0.001*	12(21%)
pN2	8 (30%)	0 (0%)		8 (13%)
**Tumor Size** (cm)#	5 (2.5–9)	5 (2.5–14)	NS	5 (2.5–14)
**Serum ALP** (mg/dl)	94 (1–330)	108 (55–495)	NS	101 (1–495)
**Serum CEA** (ng/ml)	45.4 (0.8–4598)	3.2 (0.6–84)	*p≤0.001*	7.2 (0.6–4598)
**Deaths**	20 (74%)	3 (10%)	*p≤0.001*	23 (40%)
**Median OS** (months)*	25	Not Reached	*p≤0.001*	Not Reached

* Results expressed as median (range) or

# as number of cases (percentage); NS: statistically not significant (p>.05); F: female; M: male; ALP: alkaline phosphatase; CEA: Carcinoembryonic antigen; OS: overall survival.

### Chromosomal alterations in metastatic *vs* non-metastatic sCRC

For most chromosomes analysed, sCRC with liver metastases showed similar cytogenetic profiles to those of non-metastatic tumors; this included similar (p>.05) frequencies of del(1p) (48% *vs.* 42%), polysomy of chromosome 7 (59% *vs.* 45%), del(8p) associated to gains of 8q (44% *vs.* 26%), polysomy of chromosome 13 (74% *vs.* 58%), del(18q) (52% *vs.* 32%) and gain of chromosome 20q (63% *vs.* 39%) (Table 2). The only statistically significant differences found between liver metastatic and non-metastatic sCRC were those involving chromosomes 17p (p<.001) and 22q (p = .02): all cases showing del(22q) corresponded to liver metastatic tumors (0% *vs.* 22%); del(17p13) was found in 74% of liver metastatic *vs.* 19% of non-metastatic cases; del(17p13) with a breakpoint at 17p11.2 was almost exclusively detected among sCRC with liver metastases (67% *vs.* 10%, p<.001) (Table 2), and; all except one case with del(22q) (n = 5) also demonstrated del(17p11.2) while16 cases which had del(17p11.2) did not carry del(22q). The remaining 36 tumors carried none of the two chromosomal alterations. Interestingly, whenever these two chromosomal alterations were detected, either individually or in combination, they were present in all tumor cells, suggesting they had been acquired in the ancestral tumor cell clone.

**Table 2:** Chromosomal alterations of primary tumors from liver metastatic (n = 27) versus non-metastatic sCRC patients (n = 31).

	Liver metastatic tumors (n = 27)	Non-metastatic tumors (n = 31)	*p*-value	Total cases (n = 58)
**Chromosome 1**				
Normal	7 (26%)	14 (45%)		21 (36%)
del(1p)	13 (48%)	13 (42%)	NS	26 (45%)
Polysomy	7 (26%)	4 (13%)		11 (19%)
**Chromosome 7**				
Normal	5 (19%)	14 (45%)		19 (33%)
del(7q)	5 (19%)	1 (3%)	NS	6 (10%)
q+	1 (3%)	2 (7%)		3 (5%)
Polysomy	16 (59%)	14 (45%)		30 (52%)
**Chromosome 8**				
Normal	3 (11%)	7 (23%)		10 (17%)
del(8p)	5 (19%)	4 (13%)		9 (15%)
q+	2 (7%)	3 (9%)	NS	5 (9%)
Del(8p)/8q+	12 (44%)	8 (26%)		20 (35%)
Polysomy	5 (19%)	9 (29%)		14 (24%)
**Chromosome 13**				
Normal	7 (26%)	13 (42%)	NS	20 (35%)
Polysomy	20 (74%)	18 (58%)		38 (65%)
**Chromosome 14**				
Normal	15 (55%)	19 (61%)		34(59%)
del(14q)	4 (15%)	1 (3%)	NS	5 (9%)
Polysomy	8 (30%)	11 (36%)		19 (32%)
**Chromosome 17**				
Normal	5 (19%)	20 (65%)		25 (43%)
del(17p)	20 (70%)	6 (19%)	*p*<.001	26 (45%)
Polysomy	2 (7%)	5 (16%)		7 (12%)
**Del(17p11.2)**	18 (67%)	3 (10%)	*p*<.001	21 (36%)
**Chromosmose 18**				
Normal	13 (48%)	17 (55%)		30 (52%)
del(18q)	14 (52%)	10 (32%)	NS	29 (50%)
Polysomy	0 (0%)	4 (13%)		4 (7%)
**Chromosome 20**				
Normal	5 (19%)	12 (39%)		17 (27%)
20q+	17 (63%)	12 (39%)	NS	29 (50%)
Polysomy	5 (19%)	7 (22%)		12 (21%)
**Chromosome 22**				
Normal	15 (56%)	23 (74%)		38 (66%)
del(22q)	6 (22%)	0 (0%)	*p* = .02	6 (10%)
Polysomy	6 (22%)	8 (26%)		14 (24%)

Results expressed as number of cases and percentage of cases between brackets; NS: statistically not significant (*p*>.05).

**Figure 2:**
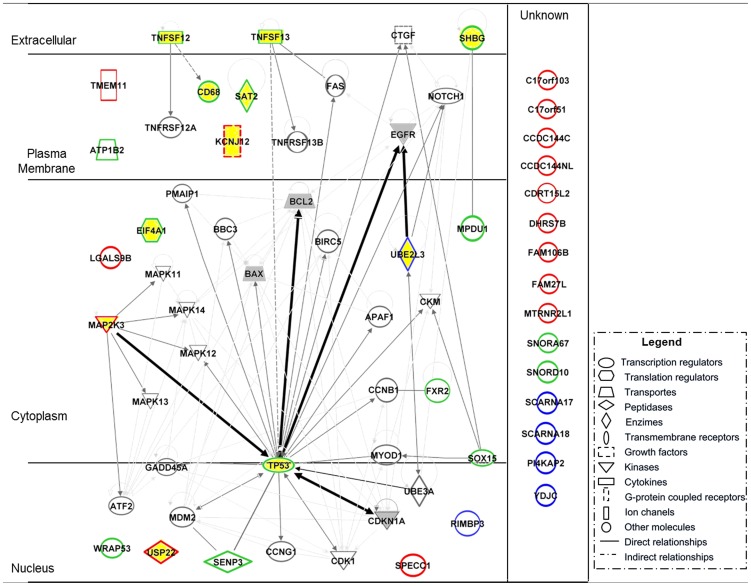
Schematic representation of the network of interactions observed between genes encoded at the 17p11.2 (genes highlighted in red), 17p13.1 (genes highlighted in green) and 22q11.2 (genes highlighted in blue) chromosomal regions, and molecules downstream molecules regulated by these genes which have been associated with cancer or cancer related signalling pathways. Genes highlighted in yellow are encoded at the three chromosomal regions referred above and they have been previously associated with cancer; genes highlighted in grey are considered as biomarkers for sCRC.

Overall, a total of 36 genes are coded at the 17p11.2, 17p13.1 and 22q11.2 chromosomal regions (Table 3); 11 out of these 36 genes (31%) have been found to be involved in cancer. The network of functional interactions among these genes and other related downstream genes implicated in cancer is depicted in Figure 2. As shown in it, such cancer-associated genes deleted in sCRC cases with del(17p11.2) and del(22q11.2) directly related to several well-established biomarkers of sCRC such as the *EGFR, BCL2, BAX* and *TP53* genes [12]–[15].

**Table 3:** List of genes encoded at chromosomal regions identified as being deleted by iFISH probes directed against the 17p11.2 (20156497 bp to 22975771 bp), 17p13.1 (7449445 bp to 7594642 bp) and 22q11.2 (21852397 bp to 21984023 bp) chromosomal regions: gene name, cell localization and function.

Coded name	Gene	Cellular localization	Function
**17p11.2**			
*C17orf103*	Chromosome 17 open reading frame 103	Unknown	Unknown
*C17orf51*	Chromosome 17 open reading frame 51	Unknown	Unknown
*CCDC144C*	Coiled-coil domain containing 144C	Unknown	Unknown
*CCDC144NL*	Coiled-coil domain containing 144 family, N-terminal like	Unknown	Unknown
*CDRT15L*	CMT1A duplicated region transcript 15-like 2	Unknown	Unknown
*DHRS7B*	Dehydrogenase/reductase (SDR family) member 7B	Unknown	Metabolism
*FAM106B*	Family with sequence similarity 106, member B	Unknown	Unknown
*FAM27L*	Family with sequence similarity 27-like	Unknown	Unknown
***KCNJ12***	Potassium inwardly-rectifying channel, subfamily J, member 12	Membrane	Transport
*LGALS9B*	Lectin, galactoside-binding, soluble, 9B	Cytoplasm	Cell-cell adhesion
***MAP2K3***	Mitogen-activated protein kinase kinase 3	Cytoplasm	Cell death
*MTRNR2L1*	MT-RNR2-like 1	Unknown	Unknown
*SPECC1*	Sperm antigen with calponin homology and coiled-coil domains 1	Nucleus	Unknown
*TMEM11*	Transmembrane protein 11	Membrane	Transport
***USP22***	Ubiquitin specific peptidase 22	Nucleus	Cell cycle
**17p13.1**			
*ATP1B2*	ATPase, Na+/K+ transporting, beta 2 polypeptide	Membrane	Metabolism
***CD68***	CD68 molecule	Membrane	Metabolism
***EIF4A1***	eukaryotic translation initiation factor 4A1	Cytoplasm	Metabolism
*FXR2*	fragile X mental retardation, autosomal homolog 2	Cytoplasm	Metabolism
*MPDU1*	mannose-P-dolichol utilization defect 1	Cytoplasm	Metabolism
***SAT2***	spermidine/spermine N1-acetyltransferase family member 2	Membrane	Metabolism
*SENP3*	SUMO1/sentrin/SMT3 specific peptidase 3	Nucleus	Metabolism
***SHBG***	sex hormone-binding globulin	Extracellular	Cell death
*SNORA67*	small nucleolar RNA, H/ACA box 67	Unknown	Unknown
*SNORD10*	small nucleolar RNA, C/D box 10	Unknown	Unknown
*SOX15*	SRY (sex determining region Y)-box 15	Nucleus	Cell differentiation
***TNFSF12***	tumor necrosis factor (ligand) superfamily, member 12	Extracellular	Cell death
***TNFSF13***	tumor necrosis factor (ligand) superfamily, member 13	Extracellular	Cell death
***TP53***	tumor protein p53	Nucleus	Apoptosis
*WRAP53*	WD repeat containing, antisense to TP53	Nucleus	Telomerase activity
**22q11.2**			
*PI4KAP2*	Phosphatidylinositol 4-kinase, catalytic, alpha pseudogene 2	Unknown	Metabolism
*RIMBP3*	RIMS binding protein 3	Nucleus	Unknown
*SCARNA17*	Small Cajal body-specific RNA 17	Unknown	Unknown
*SCARNA18*	Small Cajal body-specific RNA 18	Unknown	Unknown
***UBE2L3***	Ubiquitin-conjugating enzyme E2L 3	Cytoplasm	Metabolism
*YDJC*	YdjC homolog (bacterial)	Unknown	Metabolism

Genes which have been associated with cancer are shown in bold.

### Impact of chromosomal alterations and other disease features of liver metastatic *vs.* non-metastatic sCRC on patient overall survival

**Figure 3:**
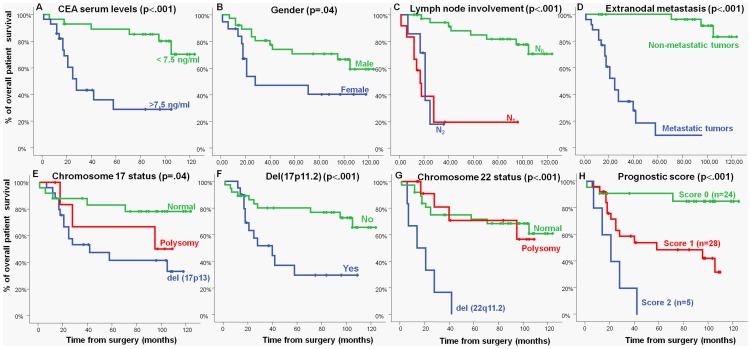
Clinical, biological and genetic characteristics of sCRC patients which showed a significant impact on overall survival in the univariate analysis: (A) carcinoembryonic antigen (CEA), (B) gender, (C) lymph node involvement, (D) occurrence of distant metastasis, (E) chromosome 17 status, (F) del(17p11.2) (G) chromosome 22 status, and (H) prognostic score established on the basis of the two most informative independent prognostic factors -del(17p11.2) and chromosome 22 status; p<.0001-.

Regarding prognosis, the presence of both del(17p13) (p = .04) -including del(17p11.2) (p<.001)- and del(22q11) (p<.001) were associated with a significantly inferior outcome. Other disease features that showed an adverse impact on patient OS were: increased (>7.5 ng/ml) CEA serum levels (p<.001), male gender (p = .04), lymph node involvement (p<.001) and, metastatic liver disease (p<.001) (Figure 3).

Multivariate analysis of the prognostic factors for OS showed that the most informative combination of independent variables to predict an adverse outcome was the presence of del(17p11.2) (p = .04) and del(22q11.2) (p = .002). Based on these two cytogenetic variables, a scoring system was built to stratify patients into a low- (no adverse features: score 0; n = 24), intermediate- (one adverse feature: score 1; n = 28) and high-risk (two adverse features: score 2; n = 5) groups with significantly different (p<.001) OS rates at 5-years: 92%, 53% and 0%, respectively (Figure 3).

### Validation of the clinical impact of del(17p11.2) and del(22q) in an independent series of patients

In order to confirm the prognostic impact of the two chromosomal abnormalities described above, we investigated their prognostic impact in an independent series of colorectal cancer patients from the public GEO database (n = 119). Noteworthy, also in this new series, patients whose tumors harboured pericentromeric breakpoints at 17p in the 17p11.2 chromosomal region (from 15 to 25 megabases from p-ter) were found to have an inferior clinical outcome than those harbouring del(17p13) alone (p = .02 and p = .04, respectively). The prognostic impact of del(17p11.2) was even stronger (p = .01) when all other tumors which showed pericentromeric deletions, including those with breakpoints in the q-arm close to the centromere (from 15 to 27.5 Mb from p-ter), were considered (Figure S1).

These results support the observations of our dataset and confirm the prognostic impact of del(17p11.2). However, the prognostic impact of del(22q) could not be confirmed (p>.05) in this new independent sCRC series of patients.

## Discussion

sCRC patients who do not show or develop distant metastasis are often cured by surgical resection of the primary tumor with optional administration of adjuvant therapy. However, when metastasis to the liver and other organs occur, the chances of cure are dramatically reduced. Despite the fact that the understanding of the genetic mechanisms underlying the early stages of both familial [16] and sporadic CRC has significantly advanced in recent years [17], the genetic mechanisms responsible for progression of sCRC to a metastatic phenotype still remain poorly understood. In this study, we investigated the pattern of numerical chromosomal alterations of primary tumors from metastatic sCRC that exhibited synchronous liver metastases versus non-metastatic sCRC. In order to avoid false-negative non-metastatic cases, in this later group only sCRC with a relative long follow up (median follow-up of 99 months) were selected for the non-metastatic tumor group. Similarly, only liver metastatic cases who had undergone complete resection of both their primary and metastatic tumor, were included in the metastatic patient group.

iFISH probes targeting those chromosomal regions more frequently altered in sCRC [6] were specifically applied to the cytogenetic characterization of both patient groups and a new probe for the definition of del(17p) associated with breakpoints at chromosome 17p11.2, was also systematically used. In line with previous observations which show that liver metastatic and non-metastatic sCRC share multiple chromosomal alterations (e.g. gains of chromosomes 7, 8q, 13q and 20q and losses of the 1p, 8p, 14q, 17p, 18q and 22q chromosomes) [7], [18]–[20], here we also found a similar distribution between liver metastatic and non-metastatic tumors for most chromosomal alterations identified. In contrast, del(22q) and del(17p) (particularly when associated with breakpoints at chromosome 17p11.2), were significantly more prevalent or even restricted, to liver metastatic tumors. These later findings support a potential role for both del(17p11.2) and del(22q) in the metastatic process of sCRC to the liver.

Previous reports based on cytogenetic analyses of metastatic disease from colorectal tumors indicated that chromosome 17p is frequently lost in sCRC [21], [22]. In line with other studies and using similar methodological approaches, our results showed the presence of del(17p13) in almost half of the sCRC cases studied [23], [24]; The frequency of del(17p13) was also significantly higher in liver metastatic than non-metastatic cases, as has been suggested by other groups [25]–[27]. It was noted that among cases with del(17p13), occurrence of a breakpoint at chromosome 17p11.2 was mostly restricted to metastatic sCRC. Coinciding with these observations, several authors have previously found that losses of chromosome 17p in metastatic CRC samples cover larger regions than in primary tumors, suggesting that unknown suppressor genes, other than the *TP53* gene, could be involved in the newly deleted 17p sequences [28]. If this is confirmed, then these differences could explain why cases with del(17p) in the absence of *TP53* mutations, also occur in advanced sCRC. Moreover, it provides evidence for the potential existence of new additional tumor suppressor genes (and potentially also oncogenes) coded in the centromeric portion of chromosome 17p, proximal to *TP53*. In this regard, it should be noted that several cancer associated genes (e.g.: *KCNJ12, MAP2K3*, and *USP22*) are coded in this chromosomal region, the first gene systematically deleted at this breakpoint region being a gene of unknown function (*FAM27L*). Interestingly, genetic polymorphisms involving this chromosomal region including the FAM27L gene, have been recently associated with an increased risk for chronic myeloid leukemia [29]. Further studies, in which mutations of this gene and deletions at chromosome 17p11.2 are searched for, may indicate their potential role in sCRC liver metastasis. Among other genes the *MAP2K3* gene is also coded in chromosome 17 region found to be commonly deleted in metastatic sCRC. *MAP2K3* is a strong promoter of tumor invasion, progression and short survival in several human cancers [30] and previous studies have shown that decreased expression of *MAP2K3* is associated with human breast infiltrating ductal carcinomas [31]; similarly, non-synonymous coding SNPs downregulating *KCNJ12* expression have been related with rhabdomyosarcomas [32], supporting a potential role for both genes in liver metastatic sCRC. However, in this chromosomal region, also some oncogenes are coded such as the *USP22* gene. Recent studies have shown that aberrant expression of USP22 is associated with liver metastasis and poor prognosis [33], due to the fact that this gene positively regulates cell cycle via both the BMI-1-mediated INK4a/ARF pathway and the Atk signaling pathway [34]. However, the activation and oncogenic role of USP22 in the progression of sCRC is potentially linked to genes encoded in other chromosomal regions such as the *BMI-1* (10p13), *CMYC* (8q24) and *CCND2* (12p13) genes [35].

In addition to del(17p11.2), in this study we also found an association between losses of chromosome 22q and disease outcome, in line with previous observations [26], [36], [37]. Previous studies based on CGH analysis [38] have shown an association between del(22q) and liver metastasis among sCRC patients; similarly, Yana *et al* [39] showed that del(22q) correlates with the Duke's stage of the disease. Iino *et al* [26] have suggested that LOH at chromosomes 17p, 18q, and 22q, is associated with an increased metastatic potential of sCRC. In the latter study, LOH at chromosome 17p was also significantly associated with vascular invasion, whereas 18q and 22q LOH correlated more with lymphatic dissemination of the disease; importantly, only LOH of chromosome 22q showed a significant association with the presence of lymph node metastasis. Thus, it could be hypothesized that in sCRC, these three chromosomal losses may be specifically associated with the metastatic process. If this holds true, screening for genetic abnormalities of primary sCRC tumors could be useful for predicting the metastatic potential which exists at the time of diagnosis [40]. It should be emphasized that analysis of del(17p11.2) in paired primary tumors and liver metastases from sCRC patients showed either presence or absence of these chromosomal changes in both (paired) tumor samples in all but two cases; in these later two cases, del(17p11.2) was only detected by SNP-arrays in the liver metastatic tumor.

Multivariate analysis of prognostic factors for OS, showed the independent prognostic value of the two chromosomal abnormalities, del(17p) with a breakpoint at 17p11.2 and del(22q); consequently, coexistence of both chromosomal alterations was associated with a significantly reduced OS *vs.* cases which showed neither of these alterations (OS at 5 years of 0% versus 93%, respectively). Despite the fact that an association has been reported between different chromosomal abnormalities and the prognosis of sCRC [18], to the best of our knowledge this is the first report in which the independent prognostic value of del(17p) with a breakpoint at 17p11.2 and of del(22q) is described. Preliminary results using genome-wide array analyses have shown an association between specific genetic alterations present in primary sCRC tumors and patient survival [10], [18], [22]. Poulogiannis *et al* (using a DNA microarray platform covering the entire genome at an average of 1 Mb of resolution) identified DNA copy number losses at 18q12.2 to be an independent prognostic marker [10]. In the current study, we have re-analyzed this dataset and confirmed the prognostic value of del(17p) including that of del(17p) with a breakpoint at 17p11.2; in contrast, the clinical impact of del(22q) could not be validated in this series. Although the precise clinical value of del(22q) should be investigated further, validation of our data concerning the prognostic impact of the 17p11.2 breakpoint in an independent dataset (in spite of the substantial differences in the technologies applied in both studies) strengthens the evidence for the clinical relevance of chromosome 17p deletions encompassing genomic regions beyond the *TP53* locus, and points to the potential role of other candidate genes coded at chromosome 17p centromericly to *TP53*. As discussed above, such genes include the *MAP2K3*, *KCNJ12* and *USP22* genes [30]–[35]. Interestingly, when we searched for direct interactions among the deleted genes and other cancer-associated genes, 30 genes deleted in cases with del(17p), and another 6 genes deleted in cases with del(22q), emerged as directly related to signaling pathways involved in cell growth and proliferation (e.g., *EGFR* and *CDK1A*) as well as in cell death (e.g., *BAX* and *BCL2*). These findings suggest a potential role for the combined deletion of these genes in conferring poor-prognosis to sCRC with coexisting del(17p) and del(22q), possibly due to increased cell proliferation and survival and diminished DNA repair.

In summary, in the present study we show that the presence of del(17p) with a breakpoint at 17p11.2 is an independent adverse prognostic factor for OS of sCRC. When combined with del(22q11.2) it allowed the identification of three groups of sCRC patients with significantly different outcome, which could be predicted at diagnosis. Further prospective studies are required in larger series of sCRC patients to confirm the prognostic value of the combined assessment of del(17p) and del(22q) in primary tumor samples at diagnosis and the precise role of the deleted genes.

## Supporting Information

Figure S1 **Validation of the impact of chromosome 17 status on overall survival in an independent series of sCRC patients from the GEO database (n = 109):** panel A, del(17p13); panels B and C, del(17p) harbouring pericentromeric breakpoints at chromosome 17p and del(17p) harboring a pericentromeric breakpoint at both chromosomes 17p and 17q, respectively.
(TIF)
